# Multicomponent Acrylic Formulation Design for Corrosion Casting with Controlled Mechanical Properties

**DOI:** 10.3390/polym15153236

**Published:** 2023-07-29

**Authors:** Pablo Reyes, Mariya Edeleva, Dagmar R. D’hooge, Ludwig Cardon, Pieter Cornillie

**Affiliations:** 1 Laboratory of Veterinary Morphology, Faculty of Veterinary Sciences, Ghent University, Salisburylaan 133, 9820 Merelbeke, Belgium; pablo.reyesisaacura@ugent.be; 2 Centre for Polymer and Material Technologies (CPMT), Department of Materials, Textiles and Chemical Engineering, Ghent University, Technologiepark 130, 9052 Zwijnaarde, Belgium; mariya.edeleva@ugent.be (M.E.); ludwig.cardon@ugent.be (L.C.); 3 Laboratory for Chemical Technology (LCT), Department of Materials, Textiles and Chemical Engineering, Ghent University, Technologiepark 125, 9052 Zwijnaarde, Belgium; 4 Centre for Textiles Science and Engineering (CTSE), Department of Materials, Textiles and Chemical Engineering, Ghent University, Technologiepark 70A, 9052 Zwijnaarde, Belgium

**Keywords:** corrosion casting, polymeric resins, polymerization

## Abstract

Corrosion casting based on the curing of acrylic resins enables one to create casts as replicas of body systems, enhancing our knowledge of veterinary medicine. The identification of the optimal chemical formulations as well as the processing conditions, the delivery of good control during the liquid state and the excellent macroscopic properties during solidification and after use are remaining challenges. In the present work, based on the identification of more qualitative trends, it is demonstrated that multicomponent comonomer mixtures are interesting materials that can be used to expand the range of mechanical properties and can specifically result in a better balance between stiffness and flexibility while guaranteeing dimensional stability. Emphasis is put on a large pool of formulations in the testing phase to then perform a detailed mechanical flexural analysis for the most promising cases during a more rigorous testing phase, accounting for a new pragmatic protocol for the pot life. This protocol consists of a vial-based turning test and a measurement of the viscosity variation up to 1000 mPa∙s and highlights the complex interplay between the overall initial concentrations and the impact of the absence of mixing once the system is at rest. It is demonstrated that the use of only low-molar-mass crosslinkers should be avoided, and overall, an intermediate amount of crosslinkers is recommendable.

## 1. Introduction

The introduction of polymers revolutionized the world as they allowed for the creation of materials suited for a large range of applications going from general to specific, e.g., from packaging to automotive parts and drug-delivery devices [1,2,3,4,5].

The current work focuses on (modifications of) poly (methyl methacrylate) (PMMA), which is a vinyl polymer playing an important role in the polymer industry [6,7,8]. Early applications of PMMA benefited from its excellent toughness and transparency, making it a suitable material for replacing glass [8,9,10,11]. A more recent PMMA commercial route is the fabrication of personal protective equipment, as boosted during the COVID pandemic [12,13,14]. PMMA is also suitable for chemical recycling due to its fast unzipping (depolymerization) tendency, increasing its circularity [15,16,17,18,19,20,21]. In the field of life sciences, its biocompatibility has made PMMA an ideal choice for being the main component in bone cement and dental resins [22,23,24,25,26,27,28,29,30,31].

Interestingly, researchers in the field of anatomy have synthesized casts based on polyacrylics, which is the larger polymer family that PMMA belongs to. A popular technique is corrosion casting, for which several polymeric resins have been investigated to create replicas of body systems, e.g., the circulatory system [32,33,34,35]. Specifically, formulations or resins with methyl methacrylate (MMA) as a key monomer component have shown to be suited for the corrosion casting of raw materials [36,37]. Such MMA-based resins (i) solidify in a reasonable amount of time, implying a reasonable pot life as the polymerization reaction is sufficiently fast; (ii) allow for a good resistance to the corroding agents (e.g., KOH, H_2_O_2_, HCl and enzymes) used to cleanse the replica from biological tissue; and (iii) are safe to handle as toxicity issues are irrelevant after full curing [32,33,38,39,40].

Previous more molecular-scale-driven research has shown that PMMA-based corrosion casting still needs to be adapted to better tune the pot life, the flow and filling performance and the durability and sustainability [32,35,36,37,40,41]. In this framework, the manufacturing of PMMA replicas is worthwhile to study both from the chemical-formulation-design and the polymer-processing-technique point of view with aims to deliver casts with more superior properties [42]. Chemical formulation design is at first sight the most interesting route for next-generation corrosion casting. Such a resin design likely requires the consideration of more complex (multicomponent) formulations providing the desired structural properties for a broader range of shapes and sizes than is currently achievable. To somewhat keep a systematic research approach and to minimize mixing issues, it is advisable to aim to obtain MMA-based formulations containing comonomers from the same monomer family, namely methacrylates, or from a very compatible sister monomer family of acrylates [43,44,45,46,47].

**Figure 1:**
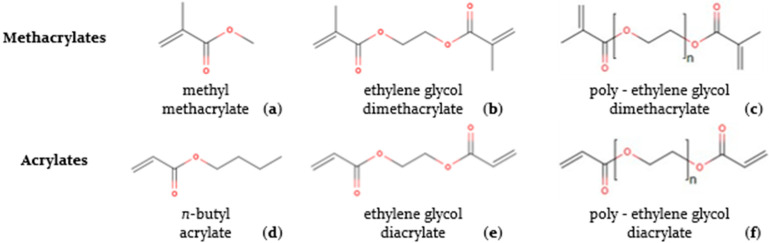
Structure of methyl methacrylate (**a**), *n*-butyl acrylate (**d**), ethylene glycol dimethacrylate (**b**), ethylene glycol diacrylate (**e**), poly-ethylene glycol dimethacrylate (**c**) and poly-ethylene glycol diacrylate (**f**).

Figure 1 displays the chemical structure of five comonomers, alongside the main monomer MMA, that are relevant for such a coformulation design, as explored in the present work; all possess a vinyl group directly attached to an ester group. A distinction is made between *n*-butyl acrylate (BA), ethylene glycol dimethacrylate (EGDMA), poly-ethylene glycol dimethacrylate (pEGDMA), ethylene glycol diacrylate (EGDA) and poly-ethylene glycol diacrylate (pEGDA). The choice of these comonomers was based on the literature data, which have shown that, for instance, BA is widely used as a comonomer to enhance flexibility and increase the impact strength [8,24,28] and to improve the adhesive properties of MMA-based coatings [48,49], bearing in mind that multifunctional monomers allow one to enhance the material strength [50]. Specifically, EGDMA and EGDA in Figure 1 have two vinyl groups, which allow them to form crosslinked systems and ultimately polymer networks to strengthen the (cast) mechanical properties [1,26,31,51,52]. To incorporate sufficient flexibility in such network polymers, it is advisable to additionally include or explore monomer building blocks with a higher molar mass [53], which explains the presence of pEGDMA and pEGDA in Figure 1.

To exploit the potential of the comonomers in Figure 1, it is important to understand the relative importance of the reactions involved and the impact of the temperature on the (propagation) reactivities. In the field of polymer reaction engineering and kinetics, most attention has been paid to understanding the mechanism behind the free radical polymerization (FRP) of MMA [54,55,56] and its copolymerization with BA [27,57,58], whereby scholars have selected a typical polymerization temperature between 343 and 373 K, although high-temperature kinetic studies also exist [59]. The leading FRP benchmarking experimental data with MMA as a monomer were recorded by Balke and Hamielec [55], showing that the initial molar ratio of the monomer to a conventional radical initiator has a huge effect on the polymerization rate and average chain length configurations, as recently modeled in full detail by De Smit et al. [15]. Furthermore, González et al. [48,49] showed that the presence of MMA reduces the branching degree in the polymer, which is otherwise dominated by BA units. Additionally, kinetic studies have been conducted for crosslinked acrylic systems, but as for the aforementioned kinetic studies, the temperature was well above the (initial) room temperature for corrosion-casting applications. For example, Bowman and Peppas studied highly crosslinked systems, choosing initiation by UV radiation [60].

A challenge thus remains to enhance the synthesis of acrylic-based formulations in a lower temperature range. Notably, in our previous work, more basic two-component monomeric formulations with MMA as the main component were already studied in combination with an accelerator to upgrade the corrosion casting. This was conducted by linking the chemical formulations with the mechanical performance of the casted (co)polymer, which allowed us to differentiate the tensile strength, flexural strength and impact strength data [42]. The formulation with 98% MMA and 2% EGDMA in the comonomer mixture was specifically interesting, as it enabled us to achieve the highest flexural strength [42]. This chemical performance correlation was supported by the launching of a novel detailed rheological protocol, which better dealt with the transition from a low-viscous to a high-viscous system during casting applications, including the dedicated averaging of individual data points.

The next logical step, being the scope of the present work, is to address the relevance of more complex multicomponent monomeric formulations based on the comonomers in Figure 1 and to identify those with an ever-better synergy than currently reachable with bicomponent monomeric combinations. In the present work, we therefore, in the first phase, scanned a broad range of novel formulations (ca. 20) and varied the mass fractions of several acrylic comonomers to then select the most interesting ones following a touch-based mechanical behavior analysis and the application of a visual inspection. In the second phase, the selected formulations (ca. 5), alongside the optimized bicomponent formulation from our previous work [42] and one commercial product (Batson’s #17 Anatomical Corrosion Kit), were subjected to more rigorous testing, including the determination of the flexural strength and modulus. It is shown that this more rigorous testing allowed us to better grasp the corrosion-casting performance properties with respect to the targeted ranges of the mechanical property values. We also enhanced the corrosion-casting field by proposing a protocol to improve the assessment of the pot life, whereby we better mimicked, under lab-scale conditions, the injection of polymer flows into large blood vessels and capillaries.

## 2. Materials and Methods

### 2.1. Chemicals

Methyl methacrylate (MMA), *n*-butyl acrylate (BA), ethylene glycol dimethacrylate (EGDMA), poly-ethylene glycol dimethacrylate (pEGDMA) and poly-ethylene glycol diacrylate (pEGDA) were purchased from Sigma-Aldrich and were provided by CHEMLAB ANALYTICAL bvba, Zedelgem, Belgium. Ethylene glycol diacrylate (EGDA) was purchased from Acros Organics and was provided by VWR International, LLC., Leuven, Belgium. Every monomer bottle, except pEGDMA, contained methyl ethyl hydroquinone (MEHQ) as a polymerization inhibitor. Monomers containing MEHQ were passed through a bed of aluminum oxide to remove this inhibitor [61]. The aluminum oxide (Al_2_O_3_; activated; neutral, Brockmann I) was purchased from Sigma-Aldrich; provided by Merck Life Science BV, Overijse, Belgium; and used as purchased. The obtained solutions were stored at 4 °C.

Benzoyl Peroxide (BPO; conventional chemical initiator), in the commercial form of Luperox^®^ A75, was purchased from Sigma-Aldrich; provided by Merck Life Science BV, Overijse, Belgium; and recrystallized from a solution in acetone [62]. *n*,*n*-Bis-(2-Hydroxyethyl)-p-toluidine (DHEPT; accelerator) was purchased from Aldrich Chemistry; provided by Merck Life Science BV, Overijse, Belgium; and recrystallized from a solution in cyclohexane-toluene (1:1) [63].

Batson’s #17 Anatomical Corrosion Kit, consisting of one 940 mL bottle of “monomer base solution”, two 100 mL bottles of “catalyst”, one 50 mL bottle of “promoter”, one 10 g bottle of “red pigment” and one 10 g bottle of “blue pigment”, was purchased from Gentaur Molecular Products, Kampenhout, Belgium.

### 2.2. Coformulations as Case in Five Groups

For every acrylic-based formulation for which the relative compositions are summarized in Table 1, the same preparation or synthesis protocol was followed. The relevant comonomers were first mixed to create a stock flask solution, which was divided into two volumes (0.8/0.2 vol.). An accelerator and initiator were added to each volume to produce the “accelerator solution” and the “initiator solution”, respectively. Mixing the two solutions started the polymerization by chemical initiation. The polymerization continued until the system reached the maximum conversion.

The formulations in this work were additionally divided into groups in Table 1 (Group I to V) to facilitate the interpretation of the casting performance, starting from the results from our previous work, whereby we already considered 14 blends to manufacture polyacrylic casts [42]. The formulations from that work had two components from the monomer-composition point of view, whereby MMA was the main component and a comonomer that was either BA, EGDMA or EGDA was the second component. The five groups together define Case A–N in Table 1, with additionally Case O producing the most promising result from our previous work (98% MMA, 2% EGMA, cM_0_/cI_0_ = 100, wA_0_/wI_0_ = 1.0; c: molar concentration; w: weight; I: initiator; M; monomer; A: accelerator). For benchmark purposes, a commercial formulation based on Batson was also considered (Case P).

The first formulation in this work (Case A in Table 1) was based on the formulation with the best results from our previous work; it had 2% of the low-molar-mass crosslinker in total. Here, the lowest contents of EGMDA (Mon_c) and EGDA (Mon_d) were employed, and thus both crosslinkers were present in the same overall amount that was similar to the historical Case O, defining the first three-component comonomer mixture. In the quest to obtain further synergies by creating variations in the comonomer mixture, three other formulations were proposed with a fourth comonomer, namely Case B, C and D in Table 1 together with Case A, which defined Group I. Case B in Table 1 included the lowest value of BA, whereby we kept the previous content of EGDMA and EGDA and sought to integrate the flexibility of the polymers based on BA (Mon_b). Case C in Table 1, with a higher content of BA and a slightly higher content of EGMA and EGDA, tried to express both extra flexibility by ca. doubling the content of BA and an increased toughness from the additional content of both crosslinkers, and it displayed a total amount that was almost double that of the previous cases. Case D in Table 1, with the lowest content of BA and higher content of EGMDA and EGDA, focused less on flexibility and more on crosslinking with a steep increase in the total crosslinker content. Every sample was prepared with either 1 or 3 min of mixing time based on a visual inspection of the clarity of the mixture.

As shown in the Results and Discussion section, Case C showed the best performance from Group I, so the following three cases in Table 1 (Case E, F and G), defining Group II, were built on that formulation. Case E in Table 1, with a higher content of BA, EGDMA and EGDA, aimed to bring Case D in Table 1, which had an increased BA content and higher amounts for both crosslinkers, closer to Case C. Case F and Case G in Table 1 were designed to only explore the effect of the ratio between the crosslinkers, namely EGDMA and EGDA. Hence, both Case F and Case G in Table 1 possessed again a higher content of BA and a higher content of crosslinkers in total, with Case F possessing a higher content of EGDMA and lower content of EGDA and Case G having the reverse situation.

Group III is defined on the base of two other formulations (Case H and I in Table 1) that were proposed to further explore the effect of the BA content. As highlighted in the Results and Discussion section, a key conclusion from investigating the previous group (Group II) was maintaining the ratio of the crosslinkers at 1:1. On these grounds, Case H is defined by the lowest content of BA with a lower amount of each crosslinker, while Case I is based on the highest content of BA and an intermediate content of each crosslinker.

For the following group of formulations, leading to Group IV being composed of Case J, K and L, both lower-molar-mass crosslinkers were substituted by similar higher-molar-mass chemical compounds, following the recommendation to have the ratio of crosslinkers at 1:1. All previous cases contained EGDMA and EGDA, and both of them possessed an intermediate chain of ethylene glycol (EG) that connected two ending groups, complemented by two methyl-methacrylate groups for EGDMA and two acrylate groups for EGDA. More in detail, the new crosslinkers pEGDMA and pEGDA differed by having several units of ethylene glycol in the intermediate chain. Case J, Case K and Case L all contained a higher BA content, and regarding the content of pEGDMA and pEGDA, Case J contained a high content of each, Case K contained the lowest content of each and Case L again contained a high content of each.

The last group of formulation cases, defining Group V in Table 1 considering Case M and Case N, was based on the combination of other crosslinker type pairs as currently explored and thus considered both low- and high-molar-mass crosslinkers. This was conducted to combine the mechanical properties conferred by the poly-(EG) crosslinkers and the ability to increase the degree of crosslinking facilitated by the smaller one-(EG)-unit crosslinkers [64], which shows that the ratio between the methyl-methacrylate and acrylate crosslinkers should be preserved. EGDMA and pEGDA were selected for the formulations of this group. Case M and N in Table 1 both contained a high content of BA, while Case M contained a slightly higher content of EGDMA and pEGDA, and Case N contained the highest content of each of these crosslinkers.

### 2.3. Initial Testing Phase for Mechanical Performance

In view of the initial testing phase, molds were fabricated for the casting of the specimens, considering a polymerizing mixture in the first stage with low viscosity (e.g., 5~9 mPa∙s) and volume shrinkage in a later stage, as described in our previous work [42]. The molds were designed to produce a rectangular prism shape that were 80 mm in length, 10 mm in width and 2 mm in depth.

For the curing of each formulation, the resin mixture was poured into the molds and left to rest for a minimum of 24 h at room temperature. Then, the shapes were removed and allowed to rest for at least 3 days on a flat surface to ensure maximum curing before proceeding further with any analysis.

The mechanical performance was, in the initial testing phase, assessed by some of the involved scientists by physically manipulating the specimens and making observations about their structural integrity, aiming to rule out those whose performance was evidently inferior. The structural integrity, through this touch-based approach, was qualitatively assessed as two parameters, namely the manual strength (MS), which is a measure of the relative amount of effort required to deform the testing bar, and the material nature (MN), which allows for a classification ranging from more brittle to more flexible. Each of these two parameters were assigned a value from 1 to 5; in the case of the MS, 1 corresponded to weak and 5 corresponded to strong, and in the case of the MN, 1 corresponded to brittle while 5 corresponded to flexible.

### 2.4. Pot Life Determination

The pot life was determined through a new protocol in the context of corrosion casting. The purpose was to use a more representative situation under lab-scale conditions for the injection and resting of the polymer flows into large blood vessels and capillaries. When casting the circulatory system, the number of possibilities in the shear rates are vast. Corrosion-casting procedures are difficult to standardize, as the material can be at rest as well as stirred, and then it can be injected. All the steps depend on the circumstances, the biological specimen to be injected and the criterion of the anatomist conducting the procedure. Hence, two opposite ends were selected, one with a high shear rate and one with no shear rate applied, being it a frame wide enough to better understand the corrosion-casting pot life.

Under the aforesaid premises, two conditions were selected: (i) at 0 s^−1^, whereby we determined the pot life in a vial with no shear rate, and (2) at 10 s^−1^, whereby we determined the pot life via a steady-state rheological measurement, under high shear rate. For condition (i), 1 mL of the mixture was poured into a 2 mL vial and kept at room temperature without agitation until we observed solidification. The inability of the mixture to flow in case the vial was turned upside down was registered as the pot life. For condition (ii), we employed the previously developed method involving rheological tests performed with an MCR 702 MultiDrive Rheometer from Anton Paar in a concentric cylinder geometry (CC) [42]. The experimentally determined viscosity vs. time data were treated via the same averaging procedure as before, which yielded the value of the pot life once the viscosity reached 1000 mPa∙s.

To understand the effect of the initiator and accelerator concentrations, their values were varied as shown in Table 2 for each selected formulation from Table 1 consisting of 6 variations.

### 2.5. Rigorous Testing Phase for Mechanical Properties

According to the manufacturing procedure mentioned in the previous subsection, silicone molds were again made to cast rectangular un-notched type 1 specimens starting from the most suited formulations from Section 2.3.

These specimens were prepared according to the ISO 179-1 standard [65]. The specimens were stabilized in a controlled temperature (23 °C) and humidity (50%) environment for at least 48 h before testing. Flexural tests were conducted according to the ISO 178 standard [66] on an Instron 4464 testing machine, using a flexion rate of 1 mm min^−1^. Additionally, in this rigorous testing phase, the dimensional stability was inspected by assessing possible relevant shrinkage.

## 3. Results and Discussion

In this section, emphasis is first put on the initial testing phase results delivering the most interesting formulations out of Table 1 that are then discussed in view of the recorded flexural properties, considering also a pot life analysis. The overall goal is the identification of the formulations with both an acceptable stiffness and flexibility, considering the comonomers in Figure 1 that were selected based on their spectrum of softer or more rigid properties, as explained in the introduction.

### 3.1. Preliminary Screening of Mechanical Performance

**Figure 2:**
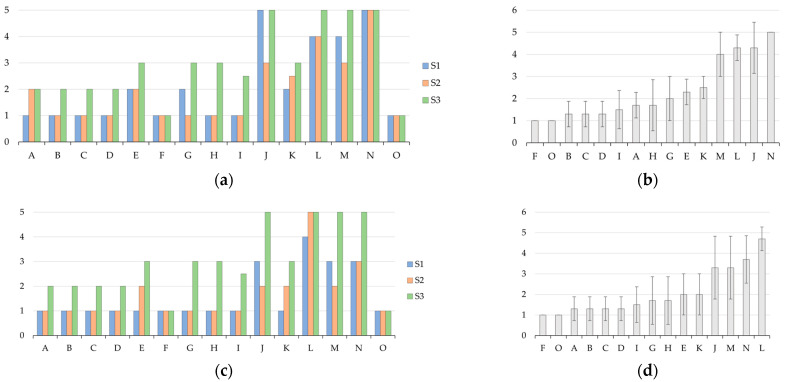
Manual strength (MS) (**top**) and material nature (MN) (**bottom**) of tested samples. On the left side, values from 3 scientists (S1–S3) with a value of 1 and 5 being weak and strong, respectively, (**a**,**c**), and on the right side, average values ordered from lowest to highest with standard deviation (**b**,**d**).

Figure 2a,c show the values assigned by three scientists to the manual strength (MS) and the material nature (MN). As described in Section 2.3, the MS is a measure of the relative amount of effort required to deform the testing bar, and the MN allows for a classification ranging from more brittle to more flexible. Those results can be further processed as average values, as shown in Figure 2b,d, for readability arranged from the lowest to the highest performance. The latter two subfigures also include standard deviation bars, allowing one to assess the degree of homogeneity between the three touch-based values. The error bar is ca. one unit, highlighting that the pragmatic approach allows for a fast scanning of the most promising candidates for further testing and that despite being prone to a certain degree of error sufficient to rule out bad formulations, time is saved for mechanical analysis testing.

The analysis of Figure 2b,d allows us to highlight that Case J and L from Group IV and Case M and N from Group V were the most interesting. They had the highest values for both the MS and MN, with values above three and even values (tending) toward five. As shown in Table 1, Group I to III (Case A to I) contained only low-molar-mass crosslinkers, while Group IV (Case J to L) contained only higher-molar-mass crosslinkers and Group V (Case M and N) contained one crosslinker of each. Noting this, it is evident that low-molar-mass crosslinkers alone are not the best option for a better mechanical performance. Supporting this statement, it follows that almost every formulation case from Group IV and Group V lied within the top performance group, with the only exception being Case K. This deviation for Case K can be understood, as it contained the lowest crosslinker content for the cases in Group IV and V. In contrast, Case J, L, M and N had a total crosslinker content that was intermediate, high, intermediate and the highest, respectively.

At first sight, low-molar-mass crosslinkers can create a more compact polymer network in comparison to those possessing a higher molar mass. However, such compactness can be overdesigned, prohibiting the request relaxation at the application and thus material level, highlighting that the scope of the present work extends the range of comonomer mixtures from bicomponent to multicomponent.

In view of general interest, Case E seemed to deliver the best performance within Group I, II and III, noting that it also contained higher crosslinker amounts, putting forward that a threshold amount of crosslinkers is needed for sure. Therefore, for the dedicated testing phase of this study, including the pot life determination, Case E, J, L, M and N were selected. Also, Case O, being the optimized historical case from our previous work [42], was included for comparison purposes, alongside Batson’s #17 (widely used commercial product [67]; Case P) for a benchmark comparison.

### 3.2. Pot Life Determination

**Figure 3:**
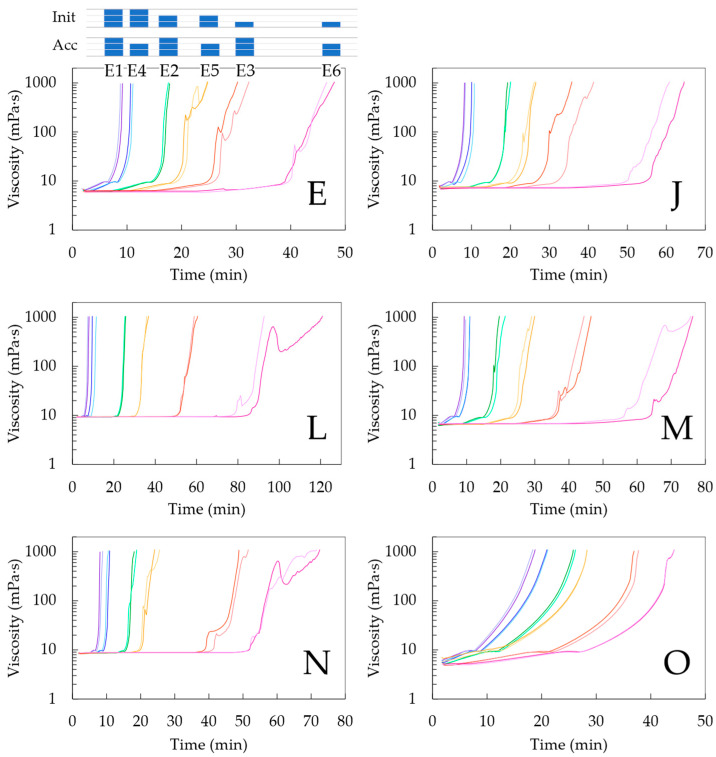
Pot life determination via rotational rheological test at a steady shear rate value of 10 s^−1^, dealing with the best 5 formulations from Table 1 based on analysis of Figure 2. More details included in Table 2 with formulations displayed from left to right indicated in purple (subcase 1), blue (subcase 4), green (subcase 2), orange (subcase 5), red (subcase 3) and magenta (subcase 6). Also, the historical Case O is included for comparison. The bars in subplot are there to guide the eye.

**Figure 4:**
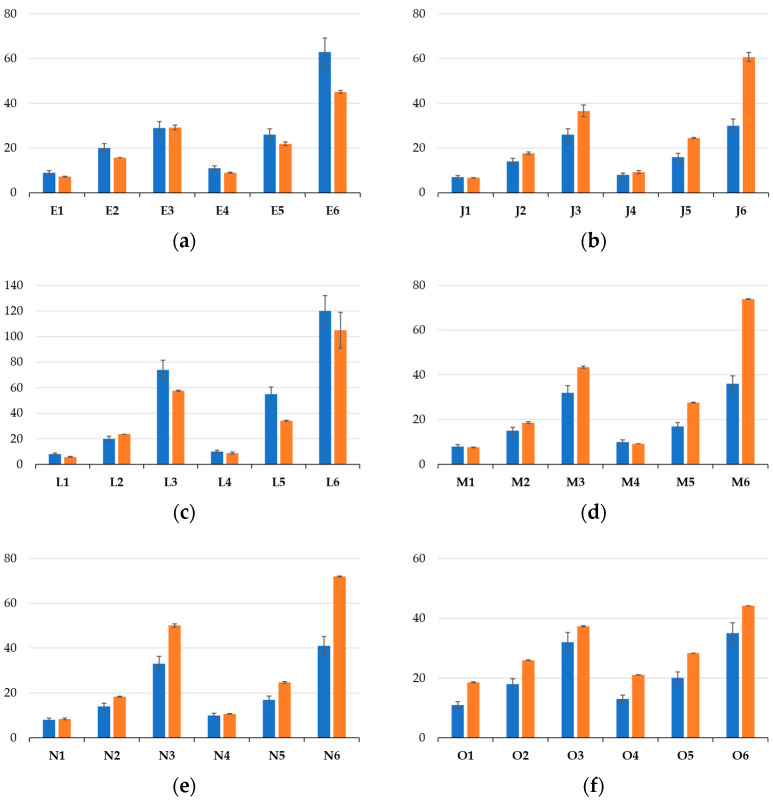
Pot life (in minutes) of mixtures determined by flask tests (blue; condition (i)) and by rotational rheology (orange; condition (ii); input from Figure 3) at a steady shear rate of 10 s^−1^ until reaching 1000 mPa∙s. Labels of *x*-axis correspond to the variations mentioned in Table 2 (**a**–**f**).

The rheometric test results for the pot life determination according to condition (ii) are shown in Figure 3. Considering the organization in Table 2 in Section 2.4, each formulation that was found to be promising from the initial testing phase (Case E, J, L, M and N) was tested at different concentrations of initiator and accelerator, with subcase 1–3 considering an increasing targeted average chain length and subcase 4–6 considering a delayed increase in this targeted average chain length due a lower relative accelerator amount. For comparison, the historical Case O was also considered. The corresponding pot lives upon reaching 1000 mPa·s, as shown in Figure 3, are also mentioned as blue bars in Figure 4.

It follows that in every subplot in Figure 3, the twelve lines can be grouped as two lines each, with each pair from left to right corresponding to an increase in the targeted chain length to a higher one. Such an increase implies a lower initial initiator concentration, causing a longer inhibition period with only a steep increase in viscosity at higher times. Within each pair, the faster increase is obtained with more accelerators present, but the effect is rather limited, making this a less critical parameter at first sight. A closer analysis of Figure 3 reveals that the lowering of the accelerator amount had, however, a more delaying effect with a higher targeted chain length. Hence, the crosslinking kinetics are strongly dependent on the variation in the initial concentrations.

In any case, it follows from Figure 3 and Figure 4 that for a given comonomer composition, one can play with the overall initial conditions of the monomer, initiator and accelerator to tune the solidification time. It should, however, be realized that the data in Figure 3 and the blue bars in Figure 4 have a given nonzero shear rate, which is more representative of the injection step of the corrosion casting and thus the lower times on the actual application level. It is thus interesting to compare these viscosities with those obtained in the absence of such a shear rate, as highlighted by the orange bars in Figure 4, whereby we selected condition (i) for the pot life determination.

It follows from these orange bars in Figure 4 that their relative position versus the blue bars is nontrivial, with both lower, equal and higher values. Although, it should be reminded that the more linear chains formed at lower conversions are prone to shear thinning, but with more crosslinking, this shear thinning effect is compensated for. On top of that, the absence of the shear rate can lead to a different mixing pattern, also showing the different reactivities of the acrylate and methacrylate moieties. Globally, it can be put forward that the largest differences are obtained with higher targeted chain lengths (subcases 5 and 6) for which the kinetics are slower, leaving more room for (statistically visible) variation.

**Figure 5:**
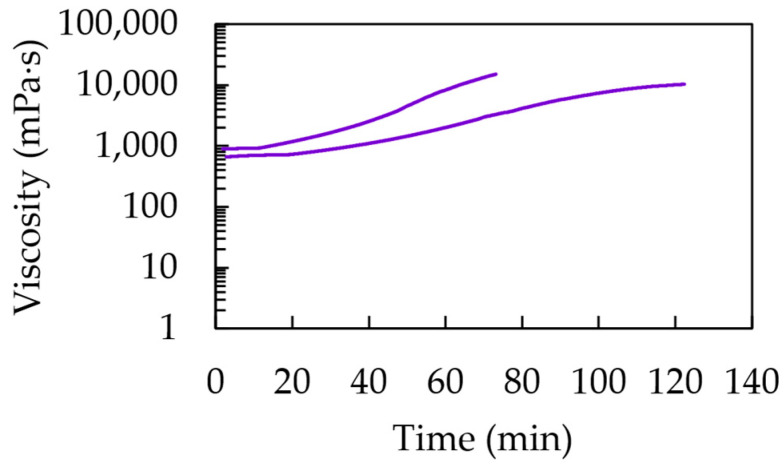
Pot life determination via rotational rheological test at a steady shear rate value of 10 s^−1^ for Case P (Batson’s #17).

For completeness, Figure 5 shows the results of the rheological test for Case P to enable the determination of the pot life of a typical commercial formulation (Batson’s #17). Here, two lines appear, reaching a final value at different times and highlighting a lesser defined reproducibility and also showing the multiaddition approach. At least one of the ingredients needs to be added in quantities as drops, which makes it less accurate and therefore more variable. Furthermore, from another experiment, the flow curve of the “monomer base solution” and hence the base resin was acquired. The data were analyzed and the viscosity value at a zero shear rate was numerically determined, yielding 1486 mPa·s. Note that a polymer was present from the start and thus the pot life should have been assessed by ignoring the 1000 mPa·s rule. Also, higher viscosities were encountered, highlighting a different type of formulation than those of the homemade formulations.

### 3.3. Rigorous Testing Phase for Mechanical Properties

**Figure 6:**
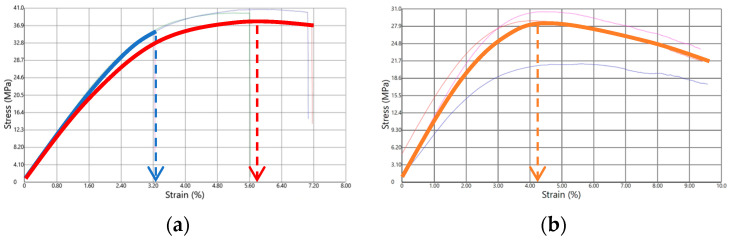
Examples of stress (MPa) vs. strain (%) curves obtained; (**a**) test with specimen breakage during the test (blue and red curves), (**b**) test in which the specimen did not break (orange curve). Arrows indicate the ultimate stress–strain values.

Upon further evaluating the casted materials selected in Section 3.1 (Case E, J, L, M, N and O) through three-points flexural testing, a more comparable mechanical dataset was collected and analyzed. Here, emphasis is on the flexural modulus (*E*_FLEX_), the maximum flexural stress (σ_MAX_) and the ultimate stress–strain (ε_fmax_). Note that the maximum flexural stress was used by identifying the maximum to determine the ultimate strain. In this context, Figure 6 shows examples of the type of stress vs. strain curves obtained. In Figure 6a, two curves are highlighted, corresponding to a material whose test specimens broke during the test, whereas in Figure 6b, the orange curve corresponds to a case in which the specimen did not break.

**Figure 7:**
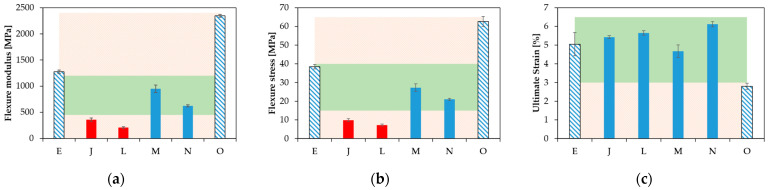
Results of the mechanical testing as (**a**) flexural modulus (*E*_FLEX_), (**b**) maximum flexural stress (σ_MAX_) and (**c**) ultimate strain (ε_fmax_). Green band indicates an optimal range, showcasing that the multicomponent design in the present work is worthwhile to access this region in all subplots, showing that the historical case is Case O. Red bars are highlighted to represent cases with the lowest stiffness.

Figure 7 summarizes the values of *E*_FLEX_, σ_MAX_ and ε_fmax_. From the group of materials tested, two cases displayed a break in their test specimens, namely Case E and O. In Case J, L, M and N, the test specimens remained, in contrast, as one piece. Case E and O also had the highest values of both *E*_FLEX_ (Figure 7a) and σ_MAX_ (Figure 7b), indicative of a relatively higher stiffness. From this criterion, Case O ranked as the formulation providing the stiffest material, followed by the formulations defined by Case E, M, N, J and L. Furthermore, upon only comparing the materials that did not break, the *E*_FLEX_ and σ_MAX_ values were the highest for M, and then in descending order, N, J and L. In addition, the ε_fmax_ value of the formulation defined by Case O was the lowest of all the materials (Figure 7c), pointing out that the maximum value of stress was achieved earlier than in any of the other cases. For ε_fmax_, however, the situation was somewhat different with the samples that did not break; the highest value corresponded to the formulations defined by Case N, and then in descending order, the formulations corresponding to Case L, J and finally M. The samples with the lowest stretchability were obtained for Case O and the highest for Case E, and globally, taking into account the larger error bar, the samples with somewhat stretchability were obtained for Case J, L and M.

**Figure 8:**
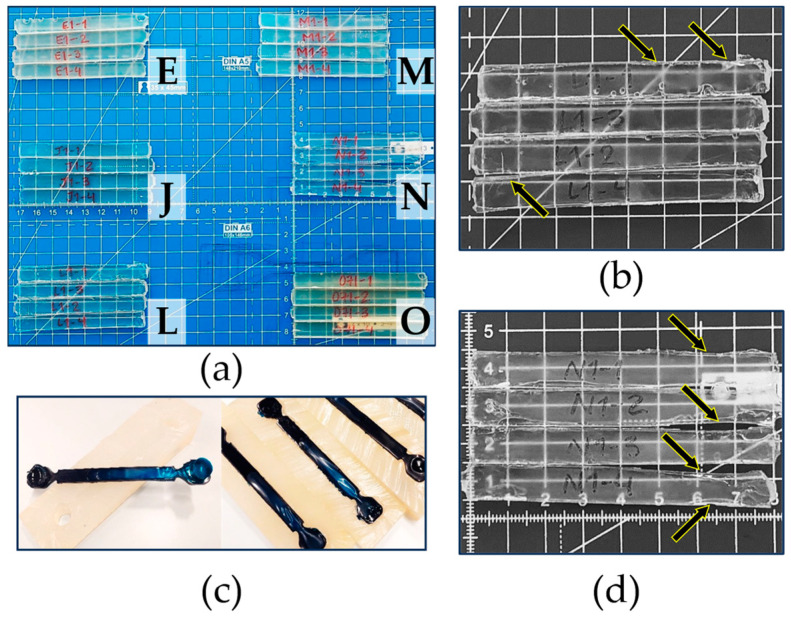
Examples of test specimens for the flexural testing in Figure 7 (**a**), details of the specimens for Case L (marked by the arrows) (**b**), test specimens for the flexural test with Case P (Batson’s #17) (**c**) and details of the specimens for Case N (marked by the arrows) (**d**).

Connecting all subplots in Figure 7, it follows that the formulation defined by Case J led to a relatively high value of ε_fmax_ coupled with a low *E*_FLEX_ and σ_MAX_, making it a very flexible material. For Case L, one can formulate similar conclusions as for Case J, with a slightly higher ε_fmax_ and also a slightly lower *E*_FLEX_ and σ_MAX_. Adding Case M and N to the comparison, the formulation defined by Case M led to somewhat higher values for both *E*_FLEX_ and σ_MAX_ and the lowest ε_fmax_ out the four materials considered. However, the ε_fmax_ values of J, L, M and N were very similar, so the latter observation was somewhat less important. Furthermore, the formulation defining Case N resulted in the highest value of ε_fmax_, and both the values of *E*_FLEX_ and σ_MAX_ were lower than those of Case M. Hence, Case N seemed to be a very interesting material, as also highlighted by the green zones in Figure 7, whereby we aimed to have all three bars in this zone and more toward the top region. However, one can also still see Case N as interesting as it had three bars in the green zone, highlighting a good compromise between stiffness and flexibility. It is mentioned for completeness that a comparison with the commercial product was, however, not possible, as important shrinkage took place upon preparing the mechanical testing bar, as shown in Figure 8c, which led to a hollow part.

Figure 8a,b,d provide additional information on the classification of the selected cases in Figure 7 through the direct visualization of the specimens cast for the mechanical testing. The test specimens made based on the formulation defined by Case E (Figure 8a in the upper-left area) and Case O (Figure 8a in the lower-right area) are the two materials associated with a much higher stiffness, so they also broke during the actual test. As explained above, the specimen from Case L (Figure 8a in the lower-left area and Figure 8b) and Case N (Figure 8a in the middle-right area and Figure 8d) should experience a reasonable mechanical performance. However, Figure 8 reveals material shrinkage after complete curing, specifically at the edges. In contrast, Case M (Figure 8a in the upper-right area) and Case J (Figure 8a in the middle-left area) both exhibited significantly less shrinkage evidence. Hence, overall, Case M was the most promising case, accounting for both mechanical properties and dimensional stability and exploiting an intermediate crosslinking amount.

Further optimization could still be worthwhile as the current work explored more qualitative trends. A beneficial research angle is the use of curing kinetic models to better understand the crosslinking degree and relation with the molar mass of the crosslinking, as recently illustrated for step-growth systems [53,64].

## 4. Conclusions

We explored novel multicomponent acrylic-based resins intended to be used as corrosion-casting formulations. We varied the composition of the resins, i.e., the amount of more rigid and flexible monofunctional comonomers and bifunctional crosslinkers as well as their nature to achieve a controlled pot life and superior mechanical performance.

We observed that the use of only more conventional low-molar-mass crosslinkers is not recommendable, and an intermediate total amount of crosslinkers is needed to ensure both a good compromise between flexibility and stiffness while ensuring a sufficient dimensional stability. The current work mainly aimed at exploring relevant, more qualitative trends, and in this respect, a further tuning of mass ratios and the use of other comonomers is recommendable to fully exploit the corrosion-casting potential, in addition to including more complex shapes.

Additionally, we put forward an improved protocol to determine the pot life, demonstrating that the initial ratio of the monomer to initiator is a key parameter.

## Figures and Tables

**Table 1:** Formulations expressed as the molar percentage of each of the six comonomers (Mon_a to Mon_f) from Figure 1 in the comonomer solution; the initiator content is expressed as the ratio of the initial molar concentration of monomer (cM_0_) to the initial molar concentration of initiator (cI_0_), and the accelerator content is expressed as the ratio of initial mass of accelerator (wA_0_) to the initial mass of initiator (wI_0_). To facilitate the comparison of cases, they are grouped in Group I–V; same table but with bars to facilitate the interpretation is in the Supplementary Materials.

Group	Case	Mon_a	Mon_b	Mon_c	Mon_d	Mon_e	Mon_f	cM_0_/cI_0_	wA_0_/wI_0_
I	A	98.0	---	1.0	1.0	---	---	100	1.0
	B	90.0	8.0	1.0	1.0	---	---	80	0.5
	C	81.0	15.0	2.0	2.0	---	---	100	1.0
	D	82.0	8.0	5.0	5.0	---	---	100	1.0
II	E	75.0	15.0	5.0	5.0	---	---	200	1.0
	F	81.0	15.0	3.0	1.0	---	---	200	1.0
	G	81.0	15.0	1.0	3.0	---	---	200	1.0
III	H	87.0	10.0	1.5	1.5	---	---	200	1.0
	I	76.0	20.0	2.0	2.0	---	---	200	1.0
IV	J	75.0	15.0	---	---	5.0	5.0	200	1.0
	K	81.0	15.0	---	---	2.0	2.0	200	1.0
	L	65.0	15.0	---	---	10.0	10.0	250	1.0
V	M	75.0	15.0	5.0	---	---	5.0	250	1.0
	N	60.0	15.0	12.5	---	---	12.5	350	0.9

**Table 2:** Initiator and accelerator content for the formulation cases tested for pot life, with the initiator content expressed as the ratio of the initial molar concentration of monomer (cM_0_) to the initial molar concentration of initiator (cI_0_) and the accelerator content expressed as the ratio of initial mass of accelerator (wA_0_) to the initial mass of initiator (wI_0_). Case letter from Table 1; same table but with bars to facilitate the interpretation in the Supplementary Materials.

Case	Subcase	cM_0_/cI_0_	wA_0_/wI_0_	Case	Subcase	cM_0_/cI_0_	wA_0_/wI_0_
E	E1	200	1	M	M1	250	1
	E2	400	1		M2	500	1
	E3	600	1		M3	750	1
	E4	200	0.7		M4	250	0.7
	E5	400	0.7		M5	500	0.7
	E6	600	0.7		M6	750	0.7
J	J1	200	1	N	N1	350	0.9
	J2	400	1		N2	525	0.9
	J3	600	1		N3	800	0.9
	J4	200	0.7		N4	350	0.7
	J5	400	0.7		N5	525	0.7
	J6	600	0.7		N6	800	0.7
L	L1	250	1	O	O1	100	1
	L2	500	1		O2	200	1
	L3	750	1		O3	400	1
	L4	250	0.7		O4	100	0.7
	L5	500	0.7		O5	200	0.7
	L6	750	0.7		O6	400	0.7

## Data Availability

Raw data on the final properties are available upon reasonable request.

## Supplementary Materials

The following supporting information can be downloaded at https://www.mdpi.com/article/10.3390/polym15153236/s1: Tables S1 and S2 with bar format for Table 1 and Table 2.
