# Supplementary material for: Multicomponent Acrylic Formulation Design for Corrosion Casting with Controlled Mechanical Properties
Source: Polymers (Basel). 2023 Jul 29;15(15):3236. doi: 10.3390/polym15153236 (PMC10422545; doi:10.3390/polym15153236)
Supplement: Supplementary file 1 [file polymers-15-03236-s001.zip › polymers-2524923-supplementary.pdf]

Supporting Information for

Multicomponent Acrylic Formulation Design for Corrosion Casting with Controlled Mechanical Properties

Pablo Reyes [1,2,3], Mariya Edeleva [2], Dagmar R. D'hooge [3,4,*], Ludwig Cardon [2] and Pieter Cornillie [1,*]

[1] Laboratory of Veterinary Morphology, Faculty of Veterinary Sciences, Ghent University, Salisburylaan 133, 9820 Merelbeke, Belgium; pablo.reyesisaacura@ugent.be
[2] Centre for Polymer and Material Technologies (CPMT), Department of Materials, Textiles and Chemical Engineering, Ghent University, Technologiepark 130, 9052 Zwijnaarde, Belgium; mariya.edeleva@ugent.be (M.E.); ludwig.cardon@ugent.be (L.C.)
[3] Laboratory for Chemical Technology (LCT), Department of Materials, Textiles and Chemical Engineering, Ghent University, Technologiepark 125, 9052 Zwijnaarde, Belgium
[4] Centre for Textiles Science and Engineering (CTSE), Department of Materials, Textiles and Chemical Engineering, Ghent University, Technologiepark 70A, 9052 Zwijnaarde, Belgium
* Correspondence: dagmar.dhooge@ugent.be (D.R.D.); pieter.cornillie@ugent.be (P.C.)

Table S1. Relative formulations of each of the six comonomers (Mon_a to Mon_f) from Figure 1 in the comonomer solution, the initiator content, and the accelerator content; all values expressed in a relative scale per column (ranging from minimum to maximum content). To facilitate the comparison of cases they are grouped in Group I-V.

Group	Case	Mon_a	Mon_d	Mon_b	Mon_e	Mon_c	Mon_f	Initiator	Accelerator
I	A	■■■■	-	■□□□	■□□□	-	-	■■■■	■■■■
	B	■■■■	■□□□	■□□□	■□□□	-	-	■■■■	■■□□
	C	■■■□	■■■□	■□□□	■■□□	-	-	■■■■	■■■■
	D	■■■□	■□□□	■■□□	■■■■	-	-	■■■■	■■■■
II	E	■■□□	■■■□	■■□□	■■■■	-	-	■■□□	■■■■
	F	■■■□	■■■□	■□□□	■□□□	-	-	■■□□	■■■■
	G	■■■□	■■■□	■□□□	■■■□	-	-	■■□□	■■■■
III	H	■■■□	■□□□	■□□□	■□□□	-	-	■■□□	■■■■
	I	■■□□	■■■■	■■□□	■■□□	-	-	■■□□	■■■■
IV	J	■■□□	■■■□	-	-	■■□□	■■□□	■■□□	■■■■
	K	■■■□	■■■□	-	-	■□□□	■□□□	■■□□	■■■■
	L	■□□□	■■■□	-	-	■■■■	■■■■	■■□□	■■■■
V	M	■■□□	■■■□	■■□□	-	-	■■□□	■■□□	■■■■
	N	■□□□	-	■■■■	-	-	■■■■	■■□□	■■■■

Table S2. Initiator and accelerator content for the formulation cases tested for pot life, with the initiator content and the accelerator content expressed in an individual relative scale (ranging from minimum to maximum content). Case letter from Table 1.

Case	Subcase	Initiator	Accelerator	Case	Subcase	Initiator	Accelerator
**E**	E1	■■■□	■■■■	**M**	M1	■■■■	■■■■
	E2	■■□□	■■■■		M2	■■■□	■■■■
	E3	■□□□	■■■■		M3	■□□□	■■■■
	E4	■■■□	■■■□		M4	■■■■	■■■□
	E5	■■□□	■■■□		M5	■■■□	■■■□
	E6	■□□□	■■■□		M6	■□□□	■■■□
**J**	J1	■■■□	■■■■	**N**	N1	■■■■	■■■■
	J2	■■□□	■■■■		N2	■■■□	■■■■
	J3	■□□□	■■■■		N3	■■□□	■■■■
	J4	■■■□	■■■□		N4	■■■■	■■■□
	J5	■■□□	■■■□		N5	■■■□	■■■□
	J6	■□□□	■■■□		N6	■■□□	■■■□
**L**	L1	■■■□	■■■■	**O**	O1	■■■□	■■■■
	L2	■■□□	■■■■		O2	■■□□	■■■■
	L3	■□□□	■■■■		O3	■□□□	■■■■
	L4	■■■□	■■■□		O4	■■■□	■■■□
	L5	■■□□	■■■□		O5	■■□□	■■■□
	L6	■□□□	■■■□		O6	■□□□	■■■□
